# Cross‐National Analysis of Consumer Preferences to Oat‐Banana‐Drinks With *Alaria esculenta* in Germany and Scotland

**DOI:** 10.1002/fsn3.72102

**Published:** 2026-07-14

**Authors:** Layla Engelhardt, Jonathan D. Wilkin

**Affiliations:** ^1^ Quality and Sensory of Plant Products Georg‐August‐Universität Göttingen Göttingen Germany; ^2^ Department of Built Environment and Life Sciences, Faculty of Social and Applied Sciences Abertay University Dundee Dundee UK

**Keywords:** algae, electronic eye, electronic tongue, Europe, novel food, sensory evaluation, TDS, UK

## Abstract

This study investigates consumer acceptance of a novel plant‐based oat‐banana drink enriched with the brown seaweed *Alaria esculenta* . To our knowledge, this is the first cross‐national comparison of such a product, combining a traditional cereal‐fruit matrix with an underutilized marine resource, evaluated in both Germany and Scotland. Sensory acceptance was assessed using a consumer study and Temporal Dominance of Sensations (TDS) analysis, while electronic eye and tongue measurements provided objective data. Principal Component Analysis (PCA) of instrumental results demonstrated a clear distinction between seaweed concentrations, supporting the sensory findings. Results indicated that the addition of up to 1% seaweed did not impair, and in some cases slightly improved, consumer acceptance. However, at concentrations of 3% and above, negative sensory attributes, particularly “green,” “fishy,”, and “unnatural,” dominated, leading to reduced liking scores. These effects were observed in both Germany and Scotland, although Scottish participants showed stronger negative associations with higher seaweed contents. Overall, the findings highlight the potential of *A. esculenta* as a functional ingredient in plant‐based beverages at low inclusion levels, while also emphasizing the importance of balancing nutritional benefits with sensory acceptance in future product development.

## Graphical abstract

Oat‐banana drinks containing 0%, 1%, 3%, or 5% edible seaweed *Alaria esculenta* were developed and evaluated by consumers in Germany and Scotland. Seaweed addition up to 1% did not adversely affect overall liking, whereas 3% and especially 5% reduced sensory acceptance. CATA profiling revealed slight regional differences: German consumers perceived the products as more uniform and natural, while Scottish consumers perceived them as more white and watery. Undesirable off‐flavors, including fishy, oily, bitter, and grassy notes, remained low across both groups, suggesting that low‐level incorporation of *Alaria esculenta* may be feasible in oat‐banana drinks.

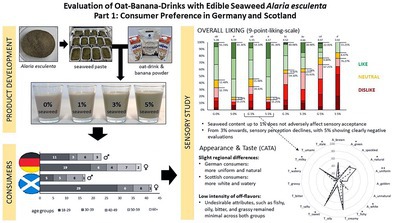

## 1 Introduction

In the contemporary context of agriculture and food science, a pivotal challenge pertains to the production of food that is both sustainable and nutritionally beneficial, while also ensuring high levels of consumer acceptance. Dietary recommendations, based on robust scientific evidence, support the consumption of a diet consisting primarily of plant‐based foods (Deutsche Gesellschaft für Ernährung (DGE) 2024; Willett et al. 2019). The consumption of plant‐based milk alternatives is increasing on a global scale. The reasons for this are diverse and often include health benefits, such as milk protein allergies, lactose intolerance, or both and consumer awareness of the environmental impact of cow's milk production (Pointke et al. 2022; Sethi et al. 2016). In order to reduce the environmental impact, it is recommended to produce and consume more regional products (Schmitt, Barjolle, et al. 2017; Schmitt, Galli, et al. 2017). In Germany and Scotland, for instance, oats ( *Avena sativa* L.) are cultivated as a regional crop, and have been a staple food for people in colder climates since ancient times. Oats are an annual crop and one of the oldest crops known to human civilization. It is cultivated for more than 2000 years in different parts of the world (Sang and Chu 2017). In 2023, Germany produced about 452,000 t of oats, ranking 11th globally, in 2024 it increased to 696.8 tons (Statistisches Bundesamt 2024). In contrast, Scotland's 2024 oat production is projected at 180,000–191,000 t, a 21% increase from 2023, contributing significantly to the UK's total of nearly one million tonnes (Scottish Goverment 2024). Oats are a nutrient‐rich crop with numerous applications. They are suitable for human consumption and have several commercial uses, including the production of oat flour and oatmeal, as well as animal feed, health products and cosmetics (Butt et al. 2008; Varma et al. 2016). Oats are a whole grain product in which all parts of the grain are processed. As a result, oat flakes and oat flour are rich in complex carbohydrates and dietary fiber, contributing to prolonged satiety and stable blood sugar levels (Salleh et al. 2019).

In contrast, seaweed has not yet established itself as a staple food in Western diets. Although it is occasionally used in traditional dishes in certain coastal regions, its consumption in countries such as Germany and Scotland remains limited. However, recent studies have indicated a rise in the consumption of seaweed in Western countries (Stévant, Rebours, and Chapman 2017; Van Den Burg et al. 2016). This phenomenon can be attributed to various factors, including globalization, migration, multiculturalism, diverse industrial applications, and an increased focus on health benefits (Fernández‐Segovia et al. 2018). Seaweed aquaculture accounts for about 97% of global seaweed production, whereas wild harvesting accounts for a negligible percentage. However, seaweed aquaculture is highly concentrated in particular regions, and the cultivation is confined to only five genera of seaweeds, with Asia as the predominant producer of aquaculture seaweed of the global production (Food and Agriculture Organization of the United Nations (FAO) and World Health Organization (WHO) 2022). A paucity of aquaculture seaweed genera, in conjunction with the highly localized nature of production areas, constitutes significant challenges for the global seaweed supply chain. Furthermore, there has been a decline in wild seaweed production of 18% between 1990 and 2019, across all three major types of seaweed classes (Food and Agriculture Organization of the United Nations (FAO) and World Health Organization (WHO) 2022). In Europe, the U.S. and Canada, seaweed farming has been particularly focused on species such as *Palmaria palmata, Alaria esculenta, Saccharina latissima*, and *Laminaria* spp., mainly for human consumption including the production of hydrocolloids such as alginates, carrageenan, and agar‐agar (Perry et al. 2019; Rioux et al. 2017). The North Sea, bordering Germany and Scotland, is a habitat for the edible brown seaweed *A. esculenta* , also known as winged kelp, one of the most frequently cultivated marine algae species in Europe, characterized by their ability to reach high biomass yield and their abundance in valuable nutritional elements (Handå et al. 2013; Kraan et al. 2000; Stévant, Marfaing, et al. 2017). Seaweeds are frugal plants which require solely carbon dioxide, light, and some nutrients in the form of hydrogen, phosphorus, and potassium to produce sufficient biomass. The Blue Economy, a rapidly expanding sector within the EU and UK, has spurred interest among many companies in sustainable ingredients with health‐promoting qualities. From 1990 to 2019, the world seaweed cultivation has increased in brown seaweed from 3.1 to 16.4 million tons and red seaweed from 1.0 to 18.3 million tons, while the green seaweed cultivation declined by approximately halved throughout the years from 31 to 17 thousand tons (Cai et al. 2021).

However, seaweeds are currently underutilized in the European food and drink manufacturing sector, while in Asian countries, the production and consumption of algae is more common (Blikra et al. 2021). The challenge of achieving consumer acceptance is much greater when these products are not part of the consumer's culinary tradition. In this case, the degree of novelty in the market would be very high, so the perceived risk could be a barrier to consuming or buying a seaweed product (Blikra et al. 2021; Palmieri and Forleo 2020). Seaweeds for human consumption in Western diets are a quite new scientific field. The acceptance is still questionable due to unpleasant sensory characteristics, such as green/blue color, fishy flavor, and off‐odors (Palmieri and Forleo 2020; Urlass et al. 2023). Undesirable odors and flavors may be masked by the addition to other foods which are already accepted by consumer or specific food processing technologies (Colonia et al. 2023; Rodríguez et al. 2024).

To create a plant‐based, regional, and health‐beneficial product to Germany and Scotland, a functional food of an oat‐banana‐drink enriched with seaweed should be tested and analyzed regarding consumer acceptance in both areas. Furthermore, our current projects align closely with industry goals and address a common challenge: consumer acceptance. To make seaweeds more acceptable in Western diets, we must thoroughly examine consumer perspectives, considering various aspects influencing their choices. Considering subjective consumer perceptions and preferences of new food sources in different cultural frameworks is important because these may differ among each other, yet cross‐national studies exploring sensory perception of food products made of seaweed are scanty. However, the additional use of electronic eye and tongue systems provides objective, reproducible measurements of key sensory attributes, complementing traditional human sensory. By combining these instrumental analyses with human evaluations, a more accurate characterization of food products and identifying sensory drivers of consumer preference, ultimately supporting the development of products that meet both nutritional and sensory expectations is possible.

In detail, this study aims to investigate the cross‐national differences between Scottish and German consumers' liking and perception of a plant‐based oat‐banana‐drink enriched with seaweed from Scotland. Specifically, the following questions should be answered: (i) How much seaweed will be accepted by consumers in the product? (ii) Which attributes describe the appearance, odor, and taste best? (iii) Are there cross‐national differences between German and Scottish consumers? and (iv) How can the product be improved?

## 2 Material and Methods

### 2.1 Oat‐Banana‐Drinks Enriched With the Brown Seaweed *Alaria esculenta*

*A. esculenta* was cultivated in a line farming system in Scotland and harvested in early autumn 2023, immediately followed by the process of dehydration and grinding into a powder. The powder was then stored under dry conditions at room temperature until utilized in this study. Briefly, 500 g *A. esculenta* powder was rehydrated with 1500 g water. In a stone grinder with double scrapper arms (Spectra 11), the seaweed and 50 g rapeseed oil were ground to a paste, adjusting the ground size every 30 min for 5 h in total. Afterwards, the paste was frozen at −20°C in small portions until use. On the day of consumption, 2000 g Alpro oat‐drink was premixed with 100 g Wholefoods banana powder. The oat‐drink was split into four aliquots enriched with 0%, 1%, 3%, and 5% seaweed (Table 1). The drinks were pasteurized (70°C ± 2°C for 2 min) to ensure microbial safety, prolong stability and provide reliable, ethically sound test samples for consumer evaluation and cooled down to 8°C. Drinks were generally used the same day as preparation, with a maximum of 2 days after they were made and stored at 8°C in the fridge.

**TABLE 1:** Recipes of the four different oat‐banana‐dinks with different concentrations of the seaweed *Alaria esculenta* , mixed *w/w*.

	Oat‐drink	Banana powder	Seaweed paste
0% seaweed drink (control)	50%	50%	0%
1% seaweed drink	99%		1%
3% seaweed drink	97%		3%
5% seaweed drink	95%		5%

### 2.2 Participants and Experimental Design of the Consumer Study

The present study was conducted in Germany and Scotland. The recruitment of consumers was done among students and employees of the University of Göttingen and Abertay University. Furthermore, only participants who did not suffer from allergies, intolerances, taste and/or smell disorders, and were not pregnant were involved in the study, in order to ensure participants' safety and to avoid biases in sensory perception and evaluation. German consumers entered their ratings using Eye Question (Elst, Netherlands), while Scottish consumers used Compusense (Guelph, Canada). Testing was conducted at Abertay University's Food Sensory Consumer labs (ISO8589:2007) and Lab for Sensory Analytics at Göttingen University (ISO8589:2007). Before starting with the first sample, participants were asked to give demographic information about gender, age, and nationality, and also if they liked to try new food products, how often they drink oat drinks, and if they have eaten seaweed products before. Data for each sample were collected in the following order: (1) check‐all‐that‐applies (CATA) ballot with 8 terms describing appearance attributes, (2) appearance liking, (3) odor liking, (4) taste liking, (5) check‐all‐that‐applies (CATA) ballot with 11 terms describing sensory attributes, (6) overall liking. All liking scores were recorded on 9 point‐hedonic scales (1 = dislike extremely to 9 = like extremely). This specific order for querying descriptive attributes and the liking ranking was chosen to obtain a more thoughtful and comprehensive hedonic assessment, rather than an immediate, spontaneous reaction. So, participants consciously processed product characteristics that may contribute to acceptance and facilitated a more comprehensive interpretation of the drivers of liking. Participants had to rinse their mouths with tap water before the first and after each sample.

### 2.3 Experimental Design of the Temporal Dominance of Sensations (TDS) Study

Panelists were recruited from within Abertay University's and Göttingen University's societal network. Per country, six panelists (3 females; 3 males) were selected using the following inclusion criteria: interest in healthy eating; a wish to try innovative food products; aged between 20 and 50 years old; non‐smokers; have good oral and general health; can distinguish between sensory attributes from a questionnaire. TDS was undertaken using the same procedure as in Ledbetter et al. (Ledbetter et al. 2025). Panelists undertook a 5 min warmup before the sensory session. During this warmup period, the concept of TDS and a definition of the attributes were explained. Panelists were encouraged to ask questions to clarify any instructions during this warmup period. Panelists were provided with 20 mL of 4 different samples, and the experiment was repeated 3 times with the same participants. This meant that each participant sampled a minimum of 12 samples.

### 2.4 Electronic Tongue

The electronic tongue α‐ASTREE Liquid Taste Analyzer (Alpha M.O.S., Toulouse, France) with a 16 sample positions autosampler and a set of seven liquid sensors (coded: AHS, PKS, CTS, NMS, CPS, ANS, SCS), for the analytics of food samples, mounted around an Ag/AgCl reference electrode was used. The e‐tongue assay required 2 min per sample, with a washing cycle of 1 min between samples. Twelve repeated measurements were made for sensor stabilization and reproducibility, and data from the last 10 measurements were used for analysis. The oat‐banana‐drinks with different *A. esculenta* concentrations were prepared from freeze‐dried material with ultrapure water (1% *w/w*), heated for 10 min for better solubility, filled in falcons, and centrifuged at 5000 rpm for 10 min at 20°C. The liquid was collected and filtered (615 ¼ filter, Macherey‐Nagel, Düren, Germany) before being analyzed.

### 2.5 Electronic Eye

The color of the oat‐banana‐drinks enriched with *A. esculenta* was assessed within the 3 different concentrations and the control without *A. esculenta* using a colorimeter IRIS (SA Alpha MOS, 31500 Toulouse, France). In a plastic petri dish, 30 mL of the sample was placed in the measuring chamber. The most widely used color space system is the L*a*b*, also known as the CIELAB system, first defined by the International Commission on Illumination in 1976, where L* is a measure of the lightness of the sample color, a* indicates red or green colors, and b* coordinate is used for yellow and blue colors.

### 2.6 Statistical Analysis

All statistical analyses were performed using R studio (version 4.4.3). Principal component analysis (PCA) and correspondence analysis (CA) were used to explore relationships among samples, sensory attributes, and consumer responses. PCA was applied to visualize patterns and variation within the sensory and hedonic data, while CA was used for the analysis and visualization of CATA attribute citation frequencies.

Overall liking scores were analyzed using a two‐way analysis of variance (ANOVA) including country (Germany and Scotland) and formulation/concentration as fixed factors. The model was used to evaluate the main effects of country and formulation as well as their interaction. When significant effects were observed, pairwise comparisons were performed using Tukey's honestly significant difference (HSD) post hoc test to identify differences between countries within the same formulation and among formulations within each country. Statistical significance was established at *p* ≤ 0.05.

CATA data were collected as binary responses indicating the presence (1) or absence (0) of each sensory attribute. Attribute citation frequencies were calculated as the percentage of consumers selecting each attribute for each sample and country group. Between‐country comparisons (Germany vs. Scotland) for individual attributes were analyzed using chi‐square tests of independence. Fisher's exact test was applied when expected cell counts were below the assumptions required for chi‐square analysis. To account for multiple comparisons, *p*‐values were adjusted using the Bonferroni correction. Correspondence analysis (CA) was performed to visualize relationships between samples and sensory attributes based on citation frequencies. Statistical significance was established at *p* ≤ 0.05. The packages “FactoMineR” (Le et al. 2008), “factoextra” (Kassambara and Mundt 2020), “ggplot2” (Wickham and Chang 2016) were used.

## 3 Results

### 3.1 Consumers´ Characteristics

A total of 120 consumers (Table 2) participated in the consumer tasting of the four variations of oat‐banana‐drinks with seaweed. In Germany, 61 consumers were recruited at the Georg‐August‐University of Göttingen, of whom 62.3% identified themselves as female and 37.7% as male. The nationalities of the consumers are listed as follows: 52 from North‐West Europe, one from South‐East Europe and Africa, one from North America, four from East Asia, two from South‐East Asia and one from East and Southeast Asia. In Scotland, 59 consumers, 71.2% female and 28.8% male participants were recruited at Abertay University in Dundee, of whom 46 consumers were of North‐West European nationality, four were of South‐East European and African nationality, one was of East Asian nationality, two were of South‐East Asian nationality, two were of South Asian nationality and four were of East and Southeast Asian origin.

**TABLE 2:** Demographic distribution of German and Scottish consumers including gender and age groups.

Participants		*N* (%)	Age groups				
			18–29	30–39	40–49	50–59	60+
**German consumers**		61 (50.8)	30	9	10	10	2
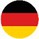	Female	38 (62.3)	19	6	4	7	2
	Male	23 (37.7)	11	3	6	3	0
**Scottish consumers**		59 (49.2)	34	11	12	2	0
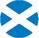	Female	42 (71.2)	29	6	6	1	0
	Male	17 (28.8)	5	5	6	1	0
**Total**		120 (100)	64	20	22	12	2
Female		80 (66.7)	48	12	10	8	2
Male		40 (33.3)	16	8	12	4	0

Consumers were asked about their interest in trying new products; in Germany 42.6% were very interested, 41.0% quite interested and only 16.4% answered “neither like nor dislike.” Similarly, in Scotland, 33.9% were very interested, 59.3% were quite interested and 6.8% answered “neither like nor dislike.” In relation to the tested oat‐banana drinks with seaweed, consumers were also asked “How often do you drink oat drinks?” The German consumer stated that 21.3% drink daily, 16.4% weekly, 16.4% monthly, 36.1% rarely and 9.8% never. The Scottish consumer said none daily, 11.9% weekly, 28.8% monthly, 39.0% rarely and 20.3% never. In each country, only one person said they had never eaten seaweed. In Germany, seaweed powder was the most commonly consumed seaweed product, followed by sushi and seaweed snacks. In Scotland, the most commonly consumed seaweed products were seaweed snacks, followed by sushi and seaweed as an ingredient. In Germany, however, 17 consumers (27.9%) did not answer.

### 3.2 Cross‐National Differences in Liking of Appearance, Odor and Taste

**FIGURE 1:**
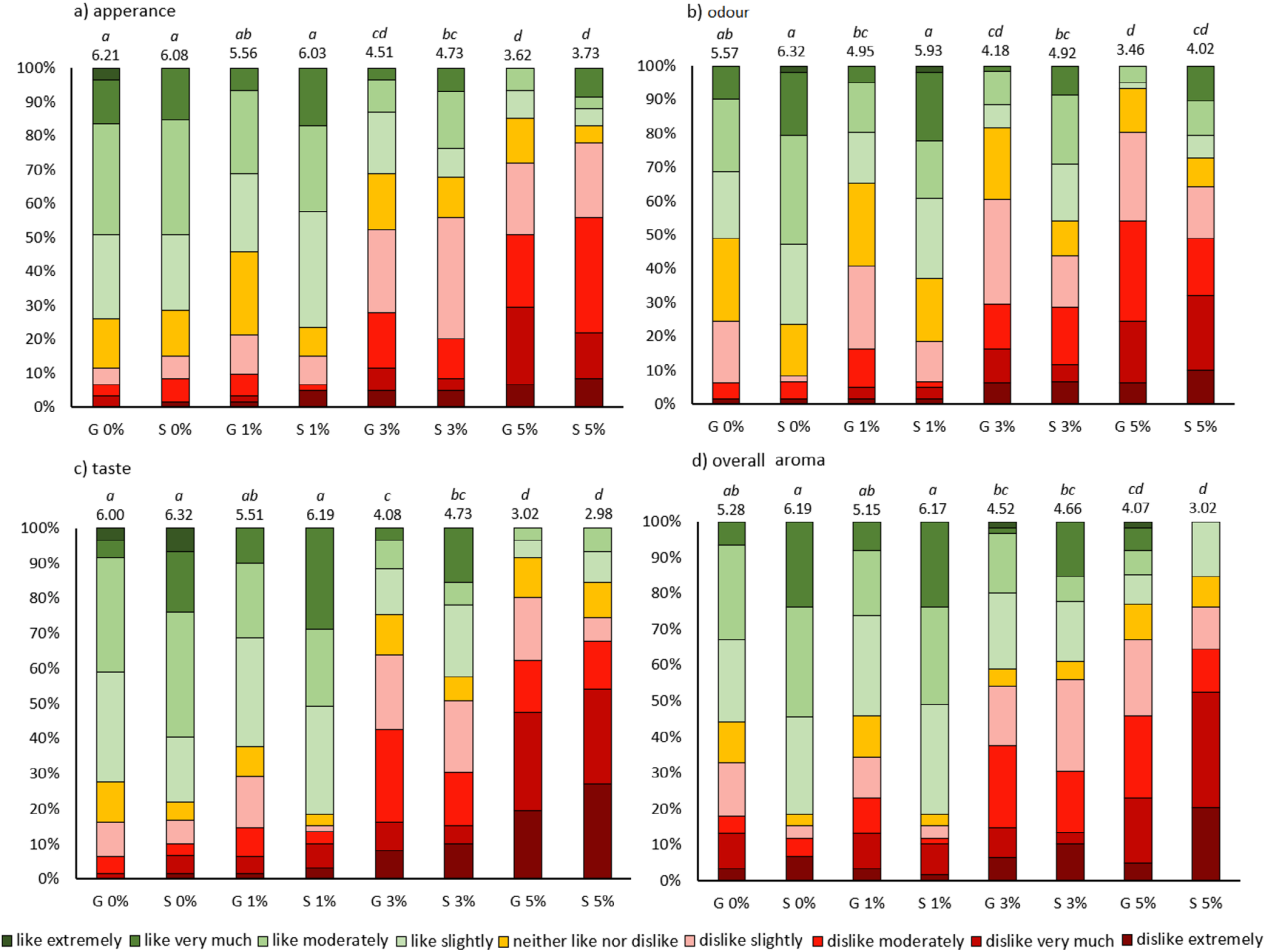
Consumer overall liking of (a) appearance, (b) odor, (c) taste, and (d) overall aroma measured on a 9‐scale from “extreme dislike” (9; dark red) over “neither nor” (5; yellow) to “extreme like” (1; dark green). Four varieties of the oat‐banana‐drink enriched with 0%, 1%, 3%, and 5% *Alaria esculenta* were evaluated in Germany (G; *n* = 61) and Scotland (S; *n* = 59). Mean values are above the bars. Significant differences (ANOVA *p* ≤ 0.05 followed by Tukey's HSD test) are marked with letters.

The overall liking shows that with increasing seaweed content the dislike of appearance, odor, taste, and overall aroma increased. Considering the drinks with the same seaweed concentration, the mean liking (Figure 1) of the appearance, odor, taste, and overall aroma did not differ between Germany and Scotland; only the aroma of the oat‐banana‐drink with 1% seaweed had a lower liking by German consumers.

No significant differences between Germany and Scotland were observed for appearance, taste, or overall aroma of the oat–banana drinks enriched with *A. esculenta* . However, odor evaluations revealed significant cross‐country differences. Specifically, for the 0%, 1%, and 3% seaweed formulations, German participants rated the odor significantly less favorably compared to Scottish participants (*p* < 0.05). As the concentration of seaweed in the drinks increases, the aversion to the drinks, indicated by the color red, simultaneously rises. The overall liking (Figure 1d) of the oat–banana drink without seaweed (0%) was rated negatively by 32.79% (b) of German and 15.25% (a) of Scottish consumers. The addition of 1% seaweed did not result in a significant change (ANOVA, *p* ≤ 0.05), with 34.43% (b) of German and 15.25% (a) of Scottish participants expressing dislike. The addition of 3% and 5% seaweed resulted in significant differences across all categories—appearance (Figure 1a), odor (Figure 1b), taste (Figure 1c), and overall aroma (Figure 1d), compared with the lower concentrations of 0% and 1% seaweed. The 3% seaweed sample was already disliked by 54.10% (bc) of German consumers and 55.93% (bcd) of Scottish consumers. A further increase in the disapproval of the product's overall liking was observed with the 5% seaweed content, with 67.21% (cd) of Germans and 76.27% (d) of Scottish consumers expressing their disapproval.

### 3.3 Objective Based Measurement of Color and Taste

**FIGURE 2:**
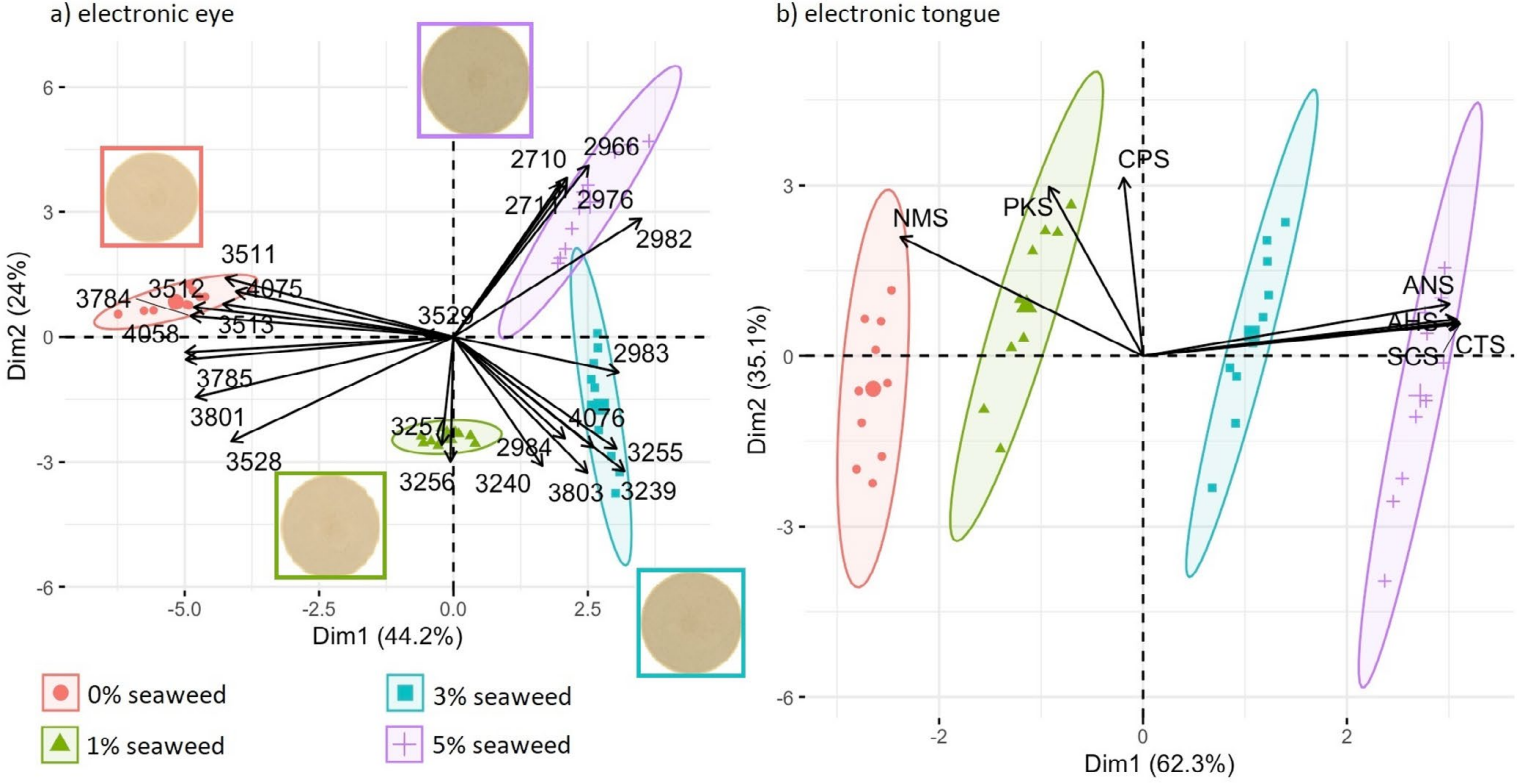
Principal component analysis of the (a) appearance measured by electronic eye (converting chart see Table 1) and (b) taste measured by electronic tongue of the oat‐banana‐drinks enriched 0% (red), 1% (green), 3% (blue), and 5% (purple) seaweed.

The electronic eye and electronic tongue were able to clearly separate the four varieties of the oat‐banana‐drinks. The appearance, measured by the electronic eye (Figure 2a), can be explained using Dim1 (44.2%) and Dim2 (24%), which accounted for a total variance of 68.2%. The 0% seaweed sample can be explained with 83.67% by the color 3512 (compare Table 3), a light yellowish brown. The 1% seaweed sample can also be explained with 9.84% by 3512, while 73.79% can be explained by 3256, a grayish yellow color. The 3% seaweed sample was 69.04% within dark grayish yellow (2983), and 10.74% a light brown olive color (2984). The 5% seaweed sample was a light brown color where 31.33% was 2967, and 21.49% a light olive brown color (2983) and 18.32% (2711), and finally 12.65% a dark grayish yellow color (2710).

**TABLE 3:** Converting electronic eye values into the CIELAB system.

No.	*L*	*a*	*b*	No.	*L*	*a*	*b*
2710	59.78	−0.99	29.6	3511	74.04	3.86	39.72
2711	60.08	1.1	20.62	3512	74.3	5.6	31.15
2966	61.47	6.37	32.1	3513	74.61	7.63	22.4
2967	61.76	8.24	23.17	3528	78.75	−3.55	36.93
2982	66.03	−3.04	37.8	3529	79.03	−1.56	28.38
2983	66.29	−1.22	29.16	3784	80.32	3.47	39.25
2984	66.6	0.95	20.28	3785	80.59	5.3	30.73
3239	67.93	5.95	31.61	3801	84.99	−3.74	36.52
3240	68.23	7.91	22.76	3802	85.28	−1.69	28.03
3255	72.44	−3.33	37.36	4058	86.8	5.04	30.34
3256	72.7	−1.41	28.75	4075	91.46	−1.79	27.7
3257	73.02	0.82	19.97	4076	91.79	0.56	19.2

Also, the e‐tongue sensors responded well to changes in the respective seaweed concentrations in the oat‐banana drinks, showing no overlap between the different seaweed concentrations (Figure 2b). The results could be explained using Dim1 (62.3%) and Dim2 (35.1%), which accounted for a total variance of 97.4%. As the seaweed concentration increased, the Dim1 scores were found to increase in the negative direction, whereas the Dim2 scores increased in the positive direction. The Dim1 was positively correlated with the sensors NMS and PKS, while CPS, ANS, SCS, and CTS were negatively correlated. In contrast, Dim2 was negatively correlated with all seven sensors. The sensors AHS and SCS showed significant differences between the samples with 0%, 1%, and 3% seaweed, but not between 3% and 5% seaweed. The sensor PKS showed a significant difference between the 1% and 5% seaweed concentrations. The sensor CTS detected significantly different values for all samples. The sensor NMS values show that the 5% seaweed sample is significantly different from the other three samples. The sensor CPS showed no differences between the samples. The sensor ANS detects differences between the 0% and 1% seaweed samples and the 3% and 5% seaweed samples.

### 3.4 Evaluation of the Appearance and Taste by German and Scottish Consumers

**FIGURE 3:**
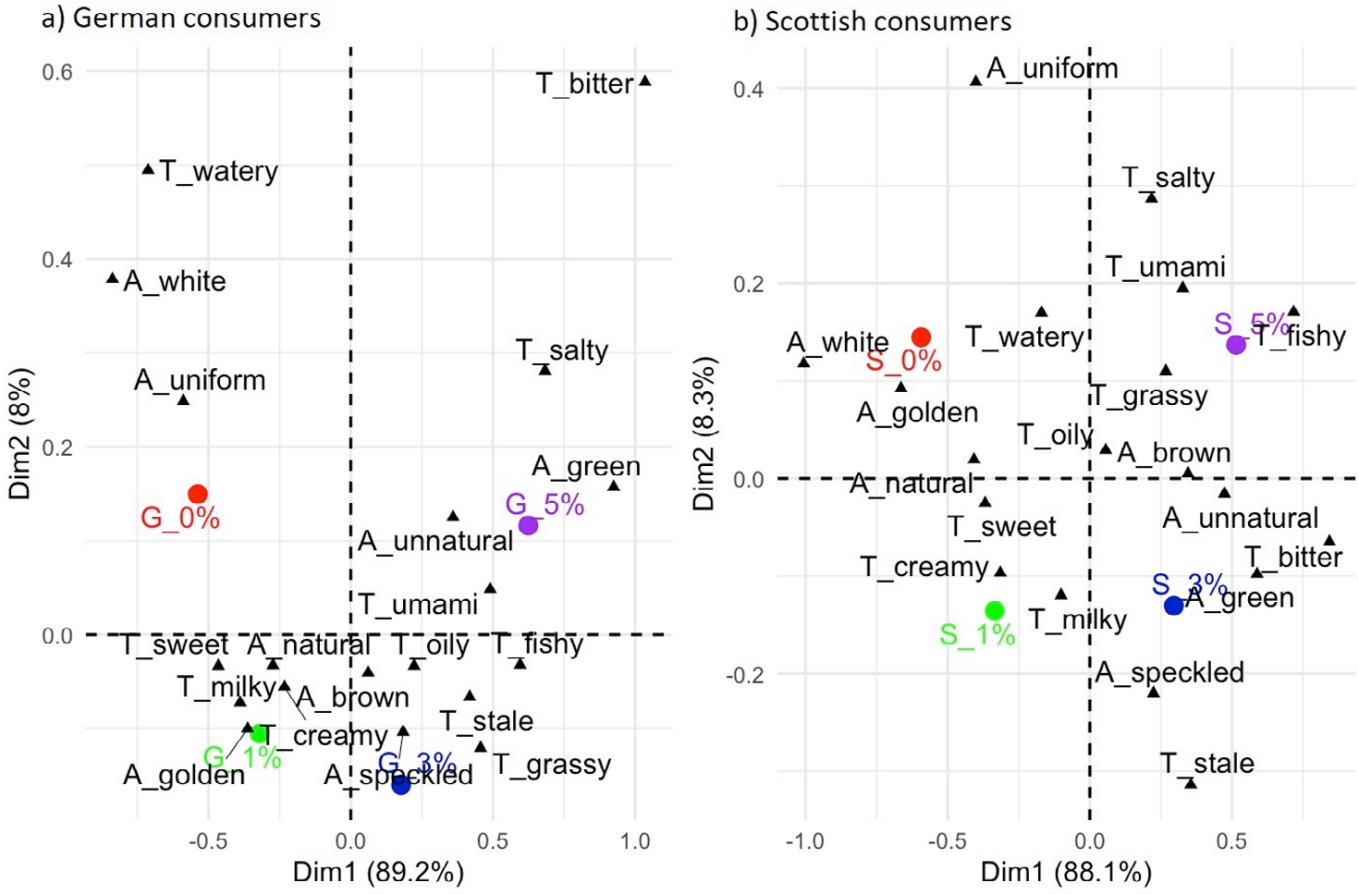
Correspondence analysis based on the appearance (A) and taste (T) CATA data from the sensory evaluation of the oat‐banana‐drinks with 0% (red), 1% (green), 3% (blue), and 5% (purple) seaweed in (a) Germany (G; *n* = 61) and (b) Scotland (G; *n* = 59).

Consumers specified their individual appearance perception out of 9 descriptors “brown,” “green,” “speckled,” “natural,” “uniform,” “golden,” “unnatural,” and “white” in a check‐all‐that‐apply (CATA) ballot. For taste perception, the 11 attributes “fishy,” “creamy,” “oily,” “sweet,” “salty,” “bitter,” “grassy,” “watery,” “milky,” “umami,” and “stale” were given. The correspondence analysis (Figure 3) of German and Scottish consumer data demonstrates that the appearance and taste attributes can be assigned to the different seaweed concentrations in the drinks. For the German consumers (Figure 3a), the total variance accounted for was 97.2%, while for the Scottish consumers (Figure 3b), the total variance accounted for was 96.4%. The correspondence analysis carried out shows clear changes in the sensory perception of the products depending on the seaweed content.

German consumers predominantly associate the product without added seaweed (0%) with neutral to slightly positive attributes such as “uniform,” “sweet,” and “natural.” Similar positive perceptions occur at a seaweed content of 1%, described as “golden,” “creamy,” and “milky.” However, as the seaweed concentration increases (3% and 5%), the products shift towards sensory attributes such as “speckled,” “grassy,” “unnatural,” and “green.” Scottish consumers also show a similar pattern: products without seaweed (0%) are described as “white,” “watery,” and “golden.” At 1% seaweed, the perception remains relatively positive and neutral. From a seaweed content of 3%, the perception clearly shifts towards “green” and “grassy.” Products with 5% added seaweed are strongly associated with negatively connoted attributes such as “fishy” and “bitter.”

The table revealed differences in appearance and taste attributes (Table 4), measured by Cohens D, of the perception of German and Scottish consumers.

**TABLE 4:** CATA descriptors of appearance (A) and taste (T) attributes selected by German (G; *n* = 61) and Scottish (S; *n* = 59) consumers in percentage (%).

	0% seaweed		1% seaweed		3% seaweed		5% seaweed	
	G	S	G	S	G	S	G	S
Appearance								
Brown	37.7%	30.5%	49.2%	40.7%	47.5%	81.4%	52.5%	86.4%
Green	1.6%	0.0%	4.9%	11.9%	27.9%	20.3%	57.4%	25.4%
Speckled	34.4%	18.6%	45.9%	52.5%	65.6%	67.8%	62.3%	54.2%
Natural	55.7%	42.4%	57.4%	37.3%	41.0%	23.7%	29.5%	17.0%
Uniform	42.6%	15.3%	27.87%	5.1%	9.8%	5.1%	9.8%	6.8%
Golden	27.9%	30.5%	24.6%	18.6%	23.0%	11.9%	8.2%	3.4%
Unnatural	6.6%	3.4%	4.9%	6.8%	9.8%	15.3%	14.8%	17.0%
White	32.8%	45.8%	14.8%	35.6%	4.9%	3.4%	1.6%	0.0%
Taste								
Fishy	11.5%	6.8%	24.6%	11.9%	59.0%	50.8%	82.0%	78.0%
Creamy	75.4%	69.5%	80.3%	78.0%	62.3%	55.9%	44.3%	33.9%
Oily	11.5%	11.9%	14.8%	11.9%	19.7%	15.3%	23.0%	15.3%
Sweet	77.0%	76.3%	68.9%	78.0%	45.9%	47.5%	18.0%	35.6%
Salty	9.8%	16.9%	6.6%	11.9%	19.7%	16.9%	47.5%	32.2%
Bitter	0.0%	0.0%	1.6%	1.7%	0.0%	28.8%	8.2%	25.4%
Grassy	9.8%	11.9%	11.5%	8.5%	36.1%	20.3%	34.4%	22.0%
Watery	14.8%	16.9%	3.3%	16.9%	3.3%	8.5%	1.6%	16.9%
Milky	63.9%	42.4%	60.7%	49.2%	45.9%	54.2%	19.7%	33.9%
Umami	3.3%	3.4%	3.3%	6.8%	8.2%	5.1%	11.5%	13.6%
Stale	9.8%	0.0%	14.8%	1.7%	29.5%	1.7%	34.4%	1.7%

CATA attribute frequencies differed between German and Scottish consumers for several sensory characteristics across the tested formulations (Table 4). At 0% seaweed, German consumers selected the attribute “uniform” significantly more frequently than Scottish consumers (42.6% vs. 15.3%, adjusted *p* = 0.037). A similar difference was observed for the 1% formulation (27.9% vs. 5.1%, adjusted *p* = 0.020). For the 3% formulation, Scottish consumers reported the attribute “brown” more frequently than German consumers (81.4% vs. 47.5%, adjusted *p* = 0.0046), and also selected the attribute “bitter” more frequently (28.8% vs. 0.0%, adjusted *p* ≤ 0.001). In contrast, German consumers more frequently described the 3% sample as “stale” compared with Scottish consumers (29.5% vs. 1.7%, adjusted *p* < 0.001). Similar trends were observed for the 5% formulation. Scottish consumers selected “brown appearance” significantly more often than German consumers (86.4% vs. 52.5%, adjusted *p* = 0.0024), whereas German consumers more frequently selected the attributes “green” (57.4% vs. 25.4%, adjusted *p* = 0.0146) and “stale” (34.4% vs. 1.7%, adjusted *p* < 0.001). Overall, the results indicate notable differences in sensory perception between German and Scottish consumers, particularly for appearance‐ and flavor‐related attributes at higher concentrations.

### 3.5 Temporal Dominance of Sensation (TDS)

**FIGURE 4:**
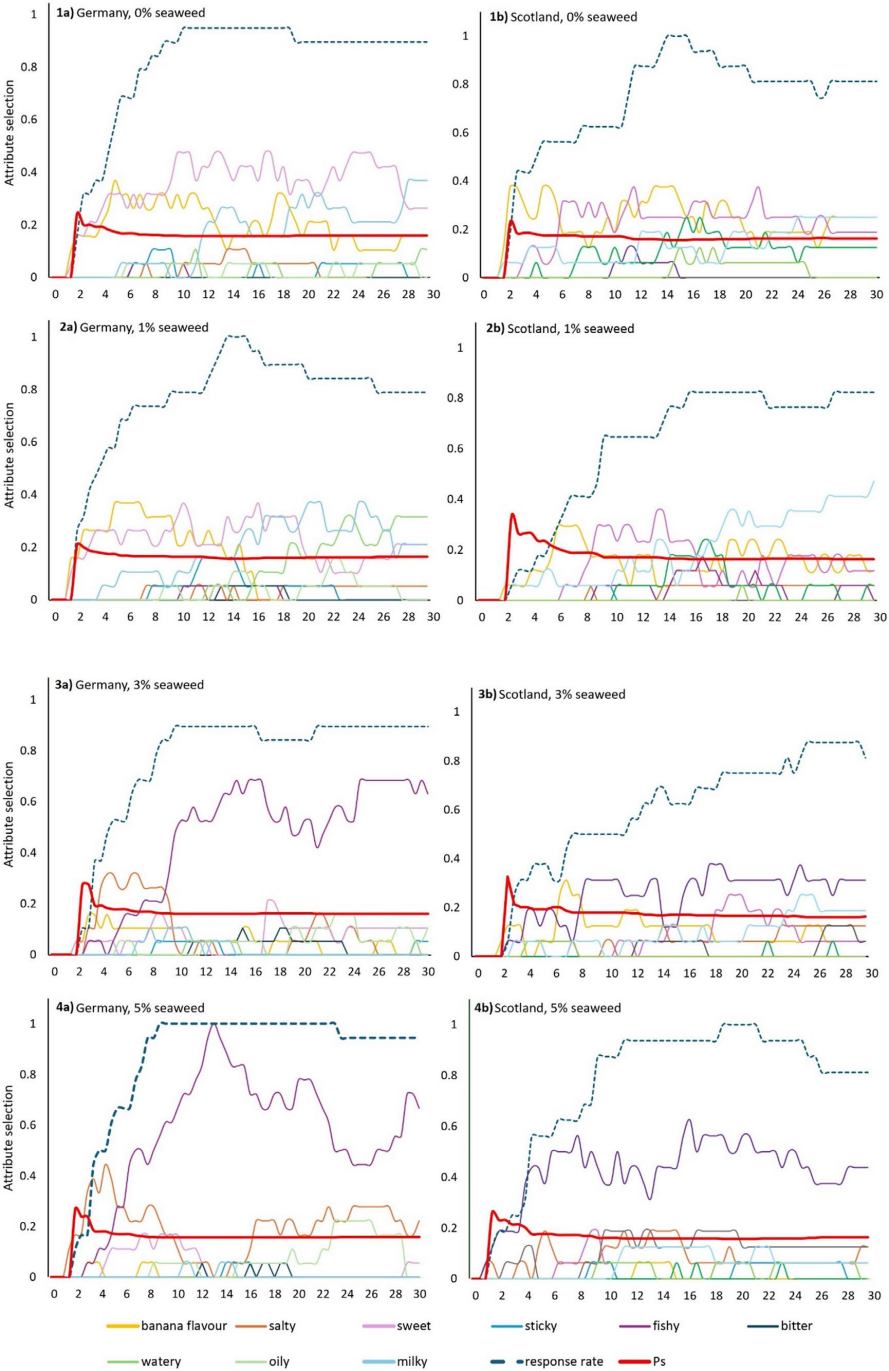
Normalized temporal dominance of sensation (TDS) curve in (1) Germany (*n* = 6) and (2) Scotland (*n* = 6) of the oat‐banana‐drinks with (A) 0% seaweed (control), (B) 1% seaweed, (C) 3% seaweed, (D) 5% seaweed. The different flavor attributes are banana flavor (yellow), salty (orange), sweet (pink), sticky (blue), fishy (purple), bitter (gray), watery (green), oily (light green), and milky (light blue). The Ps line (red) represents statistical significance (*p* ≤ 0.05), and attributes crossing this line are reported.

The typical TDS graphs (Figure 4) of oat‐banana drinks enriched with the seaweed *A. esculenta* at different concentrations and in two different countries, Germany and Scotland.

The sensory evaluation of all oat‐banana drinks with seaweed demonstrated clear disparities between the German and Scottish consumers. The dark blue stripped line represents the attribute selection, as the testing continues the attributes are selected more readily and then drops off at the end. The there is little difference to the Scottish or German cohorts when selecting attributes. The red line indicates the significance level (confidence interval), and any attribute being over this line is reported. In absence of seaweed, the perception of sweetness (pink line) remained relatively constant in the German group (Figure 4.1a) and tended to increase, while it fluctuated more strongly in the Scottish group (Figure 4.1b). A similar pattern was observed for the attribute “banana‐like” (yellow line), which shows a moderate trend in the German group (Figure 4.1a), while it is more pronounced in the Scottish group (Figure 4.1b), especially in the early phase of tasting. The “milky” attribute (light green line) also shows differences: While it is only more pronounced in Germany towards the end of the tasting, it has a certain presence in Scotland throughout the entire period. There is a noticeable difference in the perception of “watery” (dark blue line), which is much more frequent and pronounced in the Scottish group than in the German group. In the oat‐banana‐drink with 1% seaweed (Figure 4.2), the attributes “banana‐like” and “sweet” show higher intensities over time in Germany than in Scotland. The “watery” impression is also more pronounced in Scotland in certain phases of the tasting. In the oat‐banana drinks with 3% seaweed (Figure 4.3), the “fishy” note is perceived differently. While this is continuously perceived strongly in Germany, it increases in Scotland over the course of the tasting. Another striking feature is the intensity of the “salty” flavor. The salty flavor is present in both countries, although it tends to be more pronounced in Germany. Although these sweet and “banana flavor” is present in Germany, they tend to be less pronounced compared to the Scottish rating. In samples with 5% seaweed (Figure 4.4), the “fishy” note increases over time in both countries, but the overall level is higher in Germany. In Scotland, on the other hand, the “fishy” note fluctuates more strongly. The saltiness is perceived in both countries, although it is more pronounced in Germany over a longer period of time. In Scotland, on the other hand, peaks in perception occur more frequently, but at a lower level overall. Interestingly, the “oily” note is perceived more strongly and over a longer period of time in Germany, whereas it only occurs selectively in Scotland.

## 4 Discussion

The main objective of this study was to investigate consumer acceptance of different seaweed concentrations, added to an oat‐banana‐drink, following a cross‐cultural comparative approach between Germany and Scotland. All drinks, including the control samples without seaweed, were pasteurized to ensure microbiological safety and comparability across treatments. This uniform application of heat treatment means that pasteurization itself did not influence the relative sensory outcomes observed in this study. Thus, the differences in consumer perception can be attributed to the varying seaweed concentrations rather than to thermal processing. The basis was a plant‐based oat‐drink, an often‐used regional alternative to cow milk in Germany and Scotland. Often, the overall liking of plant‐based milk alternatives is rated significantly higher by vegans than by omnivores (Pointke et al. 2022), which was not asked in this study. However, German consumers stated in this study that they drink oat‐drinks more frequently than Scottish consumers. The addition of banana powder was necessary, after pre‐tests showed that the pure combination of an oat‐drink and seaweed was disliked by all asked test‐consumers. The incorporation of various regional fruits and vegetables did not result in satisfactory outcomes. By contrast, the addition of banana imparted a desirable creaminess to the oat drink, which was positively evaluated in preliminary sensory assessments. Bananas are not regional to Germany or Scotland, but they are a liked fruit in both countries, with an average consumption of around 11 kg/year/person in 2023/24 in both countries (Food and Agriculture Organization (FAO), n.d.; Statista Research Department 2025). Oat‐drinks are often used in product development to be enrich with different flavors, such as powders from freeze‐dried fruits and berries, as well as to determine their sensory and physicochemical characteristics. Oat‐drinks enriched with powders made from freeze‐dried mango, blueberries, strawberries, bilberries and raspberries had high liking by consumers (Dudarev 2024).

Overall market and consumption in the Western world are growing but remain far below Asian levels, supporting the statement that seaweed is still an uncommon food in Western countries (Young et al. 2022). The idea of using seaweed as a sustainable functional food is not new and research efforts have increased (Mohamed et al. 2012). However, the addition of seaweed to an oat‐banana‐drink was new. Not unlikely that most asked consumers, from Germany and Scotland, had eaten seaweed products before, due to the sushi boom arriving in Europe in the 1990s (Cwiertka 2005) and earlier to the USA in the 1960s (House 2018). Seaweed is mainly combined with savory products, due to the often‐used attributes of fishy and salty, such as bakery products. Also, the development of sweet products enriched with seaweed increases, even it is more difficult, due to consumers dislike of the sweet flavor combined with the fishy and salty flavor from the seaweed. Moss et al. (Moss et al. 2024) developed strawberry smoothie containing 2.5% of the brown seaweed powder *Ascophyllum nodosum* . The nutritional information and consumption information had a positive impact on the sensory perception and hedonic scores of the smoothie sample. The sustainable information led to an increased perception of fishiness in the smoothie and a decreased perception of sweetness (Moss et al. 2024). Also, a study from Portugal (Salgado et al. 2023) showed the development of three chocolates enriched with seaweed, milk chocolate with kombu, ruby chocolate with nori and white chocolate with sea lettuce, resulting in the potential of some macroalgae to be used in chocolate and this appears to be a good strategy to introduce macroalgae markets (Salgado et al. 2023).

The most important factor in product development is to find out why a product is liked or disliked, using appearance, odor, and taste of a product. Consumers from Germany and Scotland agreed regarding the liking samples with low concentrations of 0% and 1% seaweed, and disliking them with higher concentrations of 3% and 5% seaweed, which indicates that increasing concentration causes changes in appearance, odor, and taste that are perceived as unattractive by the consumers. Other studies showed that an addition of around 2.2% seaweed (Castillejo et al. 2018) and 2.5% seaweed (Moss et al. 2024) can already increase health benefits. The enrichment of soft wheat flour bread with three concentrations, 1%, 2.5%, and 4%, of the seaweeds *Chlorella vulgaris* , *Laminaria ochroleutica*, and *Arthrospira platensis* was analyzed regarding technological performance (Amoriello et al. 2021). Depending on the used algae, the addition significantly decreased water absorption and development time, while an increased stability up to 2.5% seaweed and tenacity for *L. ochroleutica* was observed. It seems that an appropriate amount of seaweed in a product might be around 2.5%, regarding health benefits, technological performance, and also consumer acceptance.

Conducting the CATA task prior to the overall liking evaluation offered the advantage of obtaining detailed sensory information that helped identify the attributes associated with consumer acceptance of the different formulations. By first encouraging consumers to reflect on specific sensory characteristics, the study was able to better relate liking responses to particular appearance, aroma, flavor, and texture attributes. This approach also facilitated interpretation of cross‐cultural differences between German and Scottish consumers by linking hedonic responses to attribute perception patterns observed in the CATA analysis. However, presenting the CATA task before the liking assessment may also have influenced consumers' hedonic judgments. Focusing participants' attention on specific sensory attributes before evaluating overall liking could increase analytical thinking during consumption and potentially reduce the spontaneity of the hedonic response. Consequently, consumers may base their liking scores more strongly on consciously perceived attributes rather than on an overall intuitive impression of the product (Ares and Jaeger 2013; Geffroy et al. 2024).

The oat‐banana‐drinks with the four seaweed concentrations can be clearly separated by color, on an objective basis by the electronic eye (Figure 2a). However, the appearance of the oat‐banana‐drinks with seaweed was rated differently between German and Scottish consumers. While green and natural aspects are perceived more strongly in Germany, brown and golden tones are more common in Scotland. In Scotland, color and texture changes are perceived as more pronounced even at lower seaweed concentrations. The perception of naturalness increases in Germany with increasing seaweed content, while this effect is less pronounced in Scotland. The speckled appearance is perceived more strongly in Scotland than in Germany. It is quite common that food enrichments lead to changes in the color of the original product. For example, an enrichment of a fermented cashew nut cheese alternative with *Chondrus crispus* and *Porphyra* sp. (Campos et al. 2024) showed significant color changes influenced by the added seaweed. The differences in the perception of the appearance might be explained by cultural differences and gastronomical contexts. Also, food neophobia is a factor which needs to be considered. A cross‐national study on crackers with algae between Italy, Belgium, Germany, and Sweden (Rabitti et al. 2024) showed that Italian consumers were significantly less food neophobic than all other countries which were, in turn, comparable. Cross‐national differences in food neophobia are prevalent among children (Proserpio et al. 2020) and adults (Ritchey et al. 2003), and they may be among the most effective predictors of consumers' acceptance of food products that have been enriched with seaweed.

The electronic tongue (Figure 2b) clearly distinguishes between the four different concentrations of seaweed in the oat‐banana‐drinks. Despite the human sense of taste consisting of five basic tastes, including sweetness, sourness, bitterness, saltiness, and umami. Consumers described in the sensory study the aroma, including odor and taste, of the oat‐banana‐drinks with 1% seaweed as having almost no fishy flavor, while the drinks with 3% seaweed were described as having an already strong fishy aroma. The occurrence of a fishy or marine odor is frequently attributable to the production of dimethyl sulphide, a compound synthesized by specific types of algae, including diatoms and dinoflagellates (Lei et al. 2018). The breakdown of sulfur‐containing compounds results in the production of dimethyl sulphide, which is responsible for the characteristic fishy or marine odor (Teng et al. 2021). Further main aroma compounds of algae and cyanobacteria cultures, with their odor threshold and olfactory descriptors, are sulfur compounds, fatty unsaturated, geosmin, aldehydes, terpenoids, and halogenated compounds (Francezon et al. 2021). Algae biomasses with high nutritional quality may be unappetizing because of unexpected flavors. Even a tiny amount of a potent odorant can affect the smell of an otherwise good product (Moran et al. 2022; Van Durme et al. 2013; Zhou et al. 2017).

Beside the information from the consumer's sensory study, a TDS study gives a valuable inside in the aroma profile of a product as well as other attributes. The profile of the oat‐banana‐drinks with seaweed showed that the flavor sweet and banana can cover slight fish flavor. From the TDS curves it's clear to see that the fishy attribute is most prevalent in the higher percentages of seaweed and it is interesting that in the lower concentration it decreased the banana taste attribute when compared to the control. The fishy flavor must be masked by the banana flavor, and combination of this at lower percentages of seaweeds. Previous studies (Wilkin et al. 2021) demonstrated significant differences in the concentration and species of seaweed used, identifying species selection as the most influential factor when conducting Temporal Dominance of Sensations (TDS) assessments with seaweed‐enhanced crackers. Ledbetter et al. (Ledbetter et al. 2025) reported that the processing methods applied to seaweed, prior to its incorporation into the cracker matrix, had a relatively minor impact compared to the quantity of seaweed used, reinforcing the importance of concentration in determining the product's sensory characteristics through TDS analysis. In contrast, the present study examines how varying concentrations of seaweed in food products, as perceived by different cohorts in Scotland and Germany, influence sensory attributes. Findings suggest that a fishy attribute is more commonly associated with formulations containing 3% or higher concentrations of seaweed. Interestingly, lower concentrations appear to enhance the perception of sweetness and other flavors, likely due to the umami and salt content, which have been previously shown to heighten consumer awareness of specific taste attributes (San Gabriel et al. 2024).

To improve a product, combining different flavors, processing and masking of unwanted flavors are options that need to be consider. Beside the seaweed concentration, which already was discussed, the banana flavor might be a piece of the puzzle to understand liking and disliking of the drink. The combination of different ingredients may also influence the acceptance of consumers. Traynor et al. (Traynor et al. 2013) paired banana and bacon, banana and olive oil, and banana and rice. The novel food pairings of banana and bacon and banana and rice were found to be liked significantly more than banana and olive oil. The results of this study suggest that synergistic and/or antagonistic interactions between the volatile compounds in the foods influenced the hedonic ratings of these food pairings. Beside the combination, also the processing method might also have an influence consumer acceptance. The study of Ledbetter et al. (Ledbetter et al. 2025) showed that *A. esculenta* had a dominant fishy flavor with no treatment, while retort fishy was decreased and after freeze thaw fishy gets more dominant. As most consumers in Western countries are unfamiliar with seaweed gastronomy, they cannot verify the attributes of flavor and texture in the products, once they are properly cooked and served (Losada‐Lopez et al. 2021). For algae products with a relatively bland smell, flavors and sweeteners may completely change the perceived flavor, and this is a practical way to develop new foods (Durmaz et al. 2020; Grossmann et al. 2020). In fact, many of the algae‐fortified products described in the literature have other flavoring agents that do not modify algae but mask their flavor (Batista De Oliveira et al. 2021; Lafarga 2019).

## 5 Conclusion

The findings of this study demonstrate that fortifying oat‐banana drinks with low levels of *Alaria esculenta* (1%–3%) holds considerable potential for product innovation in both Germany and Scotland. While the addition of 1% seaweed was well received by consumers, with overall liking scores remaining high, acceptance declined significantly at higher concentrations. Drinks fortified with 3% seaweed were already associated with sensory rejection, particularly due to negative descriptors such as “green,” “fishy,” and “unnatural.” In contrast, descriptors including “uniform,” “golden,” “white,” and “sweet” were closely linked to consumer acceptance and align with expected qualities of oat‐based beverages. These results underline the importance of carefully balancing fortification levels to achieve both sensory appeal and nutritional enhancement. To guide next steps in product development, our findings suggest that future formulations should focus on optimizing seaweed concentrations around 1%, while investigating flavor‐masking or complementary flavoring approaches to mitigate negative sensory perceptions. Additionally, strategies tailored to regional preferences may further improve acceptance in different markets. Looking ahead, further research is required to integrate consumer insights with nutritional and functional evaluations. A forthcoming second paper will therefore focus on consumers' willingness to pay, alongside a detailed analysis of mineral content and potentially health‐promoting secondary metabolites in seaweed‐fortified oat drinks. Taken together, this research trajectory will provide a comprehensive foundation for developing sustainable, acceptable, and health‐beneficial plant‐based beverages that bridge consumer demands, nutritional goals, and environmental sustainability.

## Author Contributions

**Layla Engelhardt:** conceptualization, investigation, funding acquisition, writing – original draft, methodology, validation, visualization, writing – review and editing, software, formal analysis, project administration, data curation, supervision, resources. **Jonathan D. Wilkin:** conceptualization, funding acquisition, writing – review and editing, methodology, software, formal analysis, data curation.

## Funding

The authors gratefully acknowledges funding from the European Centre of Advanced Studies (ECAS) through the 2024 Tandem Fellowship, which supported the research stay during which this work was conducted.

## Ethics Statement

The study protocol was approved by the research ethics committee of the University of Göttingen, Germany, and written consent was obtained from the individuals prior to participation, according to the declaration of Helsinki and in line with the General Data Protection regulation 2016/679.

## Conflicts of Interest

The authors declare no conflicts of interest.

## Data Availability

The data that support the findings of this study are available from the corresponding author upon reasonable request.
